# The c-Abl-RIPK3 Axis Drives Mitochondrial Dysfunction and Impaired Mitophagy in Gaucher Disease Models

**DOI:** 10.3390/antiox15040465

**Published:** 2026-04-09

**Authors:** Cristian M. Lamaizon, Renatta Tironi-Hernández, Nohela B. Arévalo, Sebastián D. Ahumada, Daniela A. Gutiérrez, Laura Brito-Fernández, Andrea del Campo, Silvana Zanlungo, Alejandra R. Álvarez

**Affiliations:** 1 Cell Signaling Laboratory, Biological Sciences Faculty, Pontificia Universidad Católica de Chile, Santiago 8331150, Chile; 2 Millennium Institute on Immunology and Immunotherapy, Biological Sciences Faculty, Pontificia Universidad Católica de Chile, Santiago 8331150, Chile; 3 Department of Gastroenterology, Medicine Faculty, Pontificia Universidad Católica de Chile, Santiago 8320165, Chile; 4 Physiology and Cell Bioenergetics Laboratory, Pharmacy School, Chemistry and Pharmacy Faculty, Pontificia Universidad Católica de Chile, Santiago 7820436, Chile

**Keywords:** Gaucher disease, lysosomal storage disorders, mitophagy, mitochondrial dysfunction, autophagy, c-Abl, RIPK3, oxidative stress

## Abstract

Gaucher disease (GD) is characterized by the accumulation of glucosylceramide within lysosomes due to mutations in the *GBA1* gene, which encodes the enzyme glucocerebrosidase. Current treatments are ineffective for patients suffering from severe neuronopathic forms of the disease. In this context, new therapeutic approaches for neuronopathic GD forms are needed. Lysosomal and mitochondrial dysfunction associated with increased oxidative stress and disturbances in the autophagic process have been described in GD. Here, we address c-Abl-RIPK3 signaling and its contribution to the accumulation of dysfunctional mitochondria in GD. Fibroblasts from patients with *GBA1* mutations and neurons treated with the glucocerebrosidase inhibitor CBE exhibited alterations in the ΔΨm and mitochondrial morphology, as well as reduced capacity to form autophagosomes. Pharmacological inhibition of c-Abl or RIPK3 restored mitochondrial function and promoted autophagosome formation, along with an increase in autophagic engulfment of mitochondria in both GD models. In conclusion, the c-Abl-RIPK3 signaling pathway contributes to mitochondrial dysfunction and blockade of autophagy components in the mitochondria, both of which are altered in the neuronopathic forms of GD.

## 1. Introduction

Lysosomal storage disorders (LSDs) constitute a group of rare disorders characterized by the accumulation of specific substrates due to reduced lysosomal enzyme activity. Among these, Gaucher disease (GD) stands out, caused by a deficiency in the enzyme glucocerebrosidase (GCase), which is responsible for hydrolyzing glucosylceramide and glucosylsphingosine [1,2]. Although GD is relatively rare in the general population, its incidence rises substantially in specific ethnic groups [3]. The *GBA1* gene, encoding GCase, harbors over 300 mutations, leading to a heterogeneous GD phenotype. GD can be broadly categorized into three clinical presentations, Type 1, Type 2, and Type 3, each associated with varying degrees of neurological involvement [4].

Type 1 is characterized by hepatosplenomegaly, cytopenia, and bone disease; it has chronic, variable severity and no primary CNS involvement, whereas Types 2 and 3 are neuronopathic forms of GD. Type 2 is characterized by an acute neuronopathic disease with severe visceral disease and early, rapidly progressive neurodegeneration, which usually has an infantile onset and high mortality. Type 3 is characterized by chronic neuronopathic involvement, presents visceral involvement plus progressive neurologic signs and slower progression than Type 2 GD, but with substantial morbidity [4].

While several treatment options have emerged for GD, there remains a significant gap in treating the neurological manifestations of Type 2 and Type 3 patients. Additionally, *GBA1* mutations associated with neuropathic forms of GD contribute to the risk of developing neurodegenerative disorders, including Parkinson’s disease (PD) and dementia with Lewy bodies [5]. These therapeutic limitations highlight the need for CNS-directed strategies that address mechanisms contributing to neuronal dysfunction.

Importantly, it has been proposed that secondary mitochondrial dysfunction is a consistent and clinically relevant feature of GD [6]. Indeed, previous studies have shown that mitochondrial dysfunction in GD models is marked by altered mitochondrial membrane potential (∆Ψm) and an increased prevalence of fragmented mitochondria [7,8,9,10,11]. This dysfunction affects oxidative phosphorylation, which is essential for meeting the energy demands and calcium buffering needs of neurons [12] and has also been associated with increased oxidative stress, as elevated levels of reactive oxygen species (ROS) and altered antioxidant responses have been reported in patient-derived cells and experimental models [13,14,15].

Mitophagy, a selective form of autophagy, plays a crucial role in maintaining mitochondrial quality control. Dysfunctional mitochondria are selectively sequestered within autophagosomes and subsequently delivered to lysosomes for degradation [16,17]. Dysregulation in these pathways can result in the accumulation of dysfunctional mitochondria and contribute to neurodegeneration [18]. In GD, mitophagy has been reported to be altered, as GCase deficiency at the lysosome impairs autophagic flux, reduces lysosomal degradative capacity, and promotes the accumulation of depolarized mitochondria [7,10,11]. However, the precise upstream mechanisms linking *GBA1* deficiency to mitophagy impairment are not well understood.

Recently, we described the activation of c-Abl kinase in several GD models [19]. Non-receptor tyrosine kinase c-Abl has been associated with various cellular functions, including the regulation of apoptosis, stress response, immune signaling, cell proliferation, and DNA repair, and has been found to be activated in various neurodegenerative diseases [20]. Importantly, c-Abl is a stress-responsive kinase that can be activated by multiple cellular stressors, including oxidative stress, DNA damage and cellular dysfunction [21,22,23]. Moreover, the activation of c-Abl has been involved in mitochondrial dysfunction, promoting fragmentation of the mitochondrial network, and contributing to a dysfunctional phenotype of mitochondria and ultimately to cell death [24,25,26,27,28].

In our previous work, we further showed that c-Abl activation in GD models leads to the activation of receptor-interacting protein kinase 3 (RIPK3) [19]. RIPK3 is a key component in necroptosis, a programmed cell death pathway that has been implicated in GD pathology [29]. In addition to its role in cell death, necroptotic signaling can modulate autophagy by affecting different steps in the autophagic process as well as autophagic machinery, which can scaffold the formation of the necrosome [30,31,31,32,33,34,35,36]. However, whether c-Abl-RIPK3 signaling contributes to mitophagy impairment and mitochondrial dysfunction in GD has not yet been investigated.

Here, we report that activation of the c-Abl-RIPK3 pathway contributes to mitochondrial dysfunction and dysregulates mitophagy in GD models by impairing the autophagy initiation of ULK1 kinase activity and autophagosome formation, along with compromised autophagic engulfment of mitochondria. Pharmacological inhibition of c-Abl or RIPK3 promotes autophagic clearance of the mitochondria. This intervention not only alleviates the observed impairments in mitochondrial health but also facilitates the labeling of dysfunctional mitochondria for autophagic clearance. These findings highlight the therapeutic potential of targeting this pathway to restore mitochondrial homeostasis in the context of Gaucher disease and other related disorders.

## 2. Materials and Methods

### 2.1. Models and Treatments

As a genetic model, we used fibroblasts obtained from the Coriell Institute, representing both healthy volunteers (GM05659) and patients with neuronopathic GD-carrying mutations in both alleles of the *GBA1* gene encoding GCase: (i) *GBA1* p.Leu483Pro (c.1448T>C; historically referred to as L444P; GM08760), which disrupts GCase folding and lysosomal trafficking, and (ii) the recombinant *GBA1* allele RecNciI, comprising p.Leu483Pro, p.Ala495Pro, and p.Val499Val (c.[1448T>C;1483G>C;1497G>C]; GM00877), known to impair enzyme stability and function [37]. These cells were maintained in DMEM with 15% fetal bovine serum (FBS) supplemented with 1× non-essential amino acids, 20 U/mL penicillin, and 20 µg/mL streptomycin and were tested for mycoplasma.

As a pharmacological model, we used rat cortical neurons treated with the GCase inhibitor, conduritol β epoxide (CBE). Cortical neurons were prepared as previously described [38]. E18 rat embryos were collected in Hank’s Balanced Salt Solution (HBSS [1 mM CaCl_2_, 40 µM MgSO_4_, 50 µM MgCl_2_, 5 mM KCl, 4 mM NaHCO_3_, 140 mM NaCl, 30 µM NaH_2_PO_4_, 40 µM KH_2_PO_4_, 6 mM D-glucose, pH 7.0]) on ice, and the cortex was dissected from the brain and collected in cold HBSS. After two washes with cold HBSS, the cells were incubated with 0.05% trypsin and 0.02% EDTA solution in HBSS at 37 °C, followed by mechanical dissociation in high-glucose DMEM supplemented with 5% horse serum via ten gentle passes through sterile Pasteur pipettes. Undissociated fragments were sedimented by brief centrifugation at 800 rpm, and the suspended cells were transferred to a new sterile tube. The recovered cells were plated in culture dishes or cover glasses previously coated with 0.25 mg/mL poly-L-lysine and maintained in high-glucose DMEM supplemented with 5% horse serum. The next day, the medium was replaced with complete Neurobasal medium (1× B27, 5 µM cytosine arabinofuranoside, 500 µM L-glutamine, 20 U/mL penicillin, and 20 µg/mL streptomycin). Primary cortical neurons were treated with CBE at 150 μM from day in vitro (DIV) 6 to DIV10.

Cells were treated for 24 h (or as indicated in the text) with the c-Abl activator DPH at 5 μM, the c-Abl inhibitor imatinib at 10 μM, or the RIPK3 inhibitor GSK’872 at 1 μM, or with 0.001% dimethyl sulfoxide (DMSO) as the vehicle. To inhibit autophagy, the autophagic flux inhibitor bafilomycin A1 (Baf A1) was added at a concentration of 100 nM or DMSO as a vehicle for 4 h. As a control for mitochondrial homeostasis disruption, cells were treated with 10 μM of the mitochondrial uncoupler carbonyl cyanide m-chlorophenyl hydrazone (CCCP) in serum-free medium for 1 h. After treatment, the cells were used for experiments.

### 2.2. ATP Levels Determination

To measure ATP levels in primary cortical neurons, cells were plated in a 96-well plate. The following day, the medium was replaced with complete Neurobasal medium, and the treatments described above were carried out. To investigate the effect of imatinib or GSK’872 on ATP levels, the inhibitors were added to the medium for 1, 4, or 24 h before the end of the experiment. As a control for decreased ATP levels, CCCP was added to the medium at a concentration of 10 µM for 1 h. ATP levels were determined using the CellTiter-Glo^®^ 2.0 Cell Viability Assay kit (Promega, Madison, WI, USA, #G9241), and luminescence was measured using the Cytation 5 instrument (BioTek Instruments, Winooski, VT, USA). ATP levels from an average of 4 technical replicates of 3 biological replicates were expressed as a percentage relative to vehicle-treated cells after subtracting the background.

### 2.3. ΔΨm-Dependent Mitochondrial Staining

To visualize mitochondria, cortical neurons or fibroblasts were treated for 30 min with MitoTracker^®^ Orange CMTMRos (ThermoFisher Scientific, Waltham, MA, USA, #M7510) at 200 nM in 1× phosphate-buffered saline (PBS): a fluorescent dye that labels mitochondria in a ΔΨm-dependent manner [39]. Then, cells were fixed with 4% paraformaldehyde (PFA) for 15 min at room temperature in the dark for immunofluorescence.

### 2.4. Immunofluorescence

Cells fixed with 4% PFA were washed twice with 1× PBS and then permeabilized with 0.3% Triton X-100 in 1× PBS (PBS-T) for 5 min. Subsequently, they were blocked with 3% bovine serum albumin (BSA) in PBS-T for 1 h and then incubated with a primary antibody against LC3B (Novus Biologicals, Centennial, CO, USA, #NB100-2220; 1:500) or against TOM20 (Santa Cruz Biotechnology, Dallas, TX, USA, #sc-11415; 1:500) overnight at 4 °C. The next day, coverslips were washed three times with 1× PBS and then incubated for 1 h at room temperature with a secondary antibody against rabbit IgG coupled to Alexa Fluor 488 (1:2000) and Hoechst 33342 (1:10,000) for fibroblasts or a primary antibody against βIII tubulin coupled to Brilliant Violet 421 (BioLegend, San Diego, CA, USA, #657412; 1:400) for cortical neurons. Coverslips were washed four times with 1× PBS for 10 min, and then they were mounted with DAKO mounting solution on glass slides.

### 2.5. Confocal Microscopy and Image Analysis

Cells were visualized using confocal microscopy on an inverted Nikon microscope with a 100× OIL Plan Apo objective NA 1.45 in the Advanced Microscopy Unit UC to capture z-stacks of 3 planes with a size of 0.1 µm on the Z-axis of each plane for fibroblasts and the soma and proximal neurites of cortical neurons. At least 5 images were acquired from 3 replicates of each experiment. Subsequently, image processing and analysis were performed using ImageJ 2.16.0/1.54p (NIH, Bethesda, MD, USA).

For fibroblasts, a custom-written macro was created to determine mitochondrial parameters. For ΔΨm, the median projection of a Mitotracker or TOM20 signal was generated on the Z-axis, and a median filter with a radius of 1 pixel and a top-hat filter with a radius of 5 pixels were applied to normalize mitochondrial labeling. Subsequently, a mask was generated on this image with a threshold value defined by the Triangle algorithm, and the average fluorescence intensity of Mitotracker was measured on the original image after Z-axis projection. Additionally, by analyzing particles, the number of mitochondria per cell and their individual area were calculated, and for each biological replicate, the percentage of cells displaying a fragmented mitochondrial phenotype was determined. For each cell, a maximum-intensity Z-projection was generated with the TOM20 channel and then classified in a blinded manner as either fragmented when mitochondria appeared predominantly punctated and discontinuous or fused/intermediate when mitochondria formed elongated, interconnected tubular networks or a mixture of morphology throughout the cell cytoplasm. For cortical neurons, a mask of the soma was generated using the βIII tubulin signal, and Mitotracker intensity was measured in the soma.

To assess colocalization between LC3 and Mitotracker in proximal neurites, since LC3 and Mitotracker signals are discrete, the degree of object-based colocalization between LC3 and Mitotracker was determined as the percentage of mitochondria positive for LC3 after applying a binary mask in a 30 µm segment of the neurite for both signals.

### 2.6. Expansion Microscopy and Image Analysis

Cells fixed in 4% PFA were washed twice with 1× PBS and permeabilized in 0.3% PBS-T for 5 min. Following permeabilization, samples were blocked in 3% BSA and prepared in PBS-T for 2 h at room temperature. Then, cells were incubated with primary antibodies against c-Abl (Millipore, Burlington, MA, USA, #A5844, 1:200) and TOM20 (Santa Cruz Biotechnology, Dallas, TX, USA, #sc-11415, 1:200) overnight at 4 °C. After three washes with 1× PBS, cells were incubated with secondary antibodies coupled to goat Alexa Fluor 488 anti-mouse (for c-Abl) and goat Alexa Fluor 555 anti-rabbit (for TOM20) at a 1:200 dilution in blocking buffer overnight at 4 °C and protected from light. Samples were washed three times in 1× PBS and then incubated with 1 mM methacrylic acid N-hydroxysuccinimide ester in 1× PBS overnight at room temperature. Gels were prepared with a gelation solution (900 mM sodium acrylate, 350 mM acrylamide, 100 mM N,N′-methylenebisacrylamide, 2 M NaCl, 580 mM 4-hydroxy-TEMPO, 0.2% m/v ammonium persulfate, 0.2% TEMED, and 1× PBS) directly on coverslips by incubating 40 µL of gelation solution between a parafilm and coverslips for 1 h at 37 °C in a humified chamber, and subsequently, gels were digested with proteinase K solution (8 U/mL proteinase K, 0.05% triton X-100, 1 mM EDTA, 50 mM Tris-HCl, and 1M NaCl) under gentle agitation overnight at room temperature and protected from light. To achieve isotropic expansion, gels were washed three times in 1× PBS and then expanded by three sequential incubations in deionized water (30 min each, as a minimum). The expansion factor of each gel was determined as the total area after expansion relative to the total area of the coverslip. Expanded gels were mounted on poly-L-lysine (PLL)-coated glass-bottom dishes with the cellular side facing the glass and were imaged on an inverted Nikon microscope with a 60× OIL Plan Apo objective NA 1.40 in the Advanced Microscopy Unit UC. Z-stacks were acquired with a 1 µm step size covering the full cellular volume.

Image analysis was performed in ImageJ 2.16.0/1.54p (NIH, Bethesda, MD, USA) using a custom-written macro to quantify c-Abl clustering and its colocalization with mitochondria. First, voxel dimensions were calibrated according to the experimentally determined expansion factor (see above). The macro then separated the c-Abl and TOM20 fluorescence channels, applied a 3D smoothing filter, and generated maximum intensity projections to facilitate thresholding. After interactive threshold selection, binary masks were created for c-Abl and mitochondria. The logical operation ‘AND’ was used to distinguish c-Abl localized within mitochondria from non-mitochondrial c-Abl. Using the “3D Objects Counter” plugin, the macro quantified the number of c-Abl clusters normalized to the sample volume, as well as the mean of cluster volumes. In addition, the macro calculated the ratio of mitochondrial to non-mitochondrial c-Abl, which reflects the relative enrichment of c-Abl within mitochondrial regions compared to the rest of the cell, serving as a quantitative indicator of c-Abl association or recruitment to the mitochondria, and the percentage of mitochondrial c-Abl relative to the total TOM20 volume, providing the proportion of mitochondrial volume that is occupied by c-Abl and indicating the extent of mitochondrial coverage by c-Abl.

### 2.7. Western Blot

For protein extraction, cells were washed with cold 1× PBS and lysed in a radioimmunoprecipitation assay (RIPA) buffer with a cocktail of phosphatase and protease inhibitors. The lysates were incubated for 30 min on ice and then centrifuged at 10,000 rpm for 10 min at 4 °C. The supernatant was collected, and protein concentration was measured using the Pierce™ BCA Protein Assay Kit (Thermo Fisher Scientific, Waltham, MA, USA). Electrophoresis was performed on a 10% polyacrylamide gel for medium-to-heavy-weight proteins and 12% or 15% gel for medium-to-light-weight proteins, with 30 µg of protein in the loading buffer. Proteins were transferred to a PVDF membrane with a pore size of 0.2 µm. The membrane was blocked in 3% BSA or non-fat dry milk (NFDM) in TBS-T for 1 h at room temperature with agitation. Subsequently, the membrane was incubated overnight at 4 °C with a primary antibody against the protein of interest (anti-LC3B antibody [Cell Signaling Technology, Danvers, MA, USA, #2775S], anti-SQSTM1/p62 antibody [Abcam, Cambridge, UK, #ab56416], anti-c-Abl phosphorylated at Y412 antibody [Millipore, Burlington, MA, USA, #C5240], anti-c-Abl antibody [Millipore, Burlington, MA, USA, #A5844], anti-RIPK3 phosphorylated at T231 and S232 antibody [Abcam, Cambridge, UK, #ab205421], anti-RIPK3 antibody [Novus Biologicals, Centennial, CO, USA, #NBP1-77299], anti-ULK1 phosphorylated at S317 antibody [Cell Signaling Technology, Danvers, MA, USA, #37762], anti-ULK1 antibody [Cell Signaling Technology, Danvers, MA, USA, #8054], anti-GAPDH antibody [Santa Cruz Biotechnology, Dallas, TX, USA, #sc-32233], anti-actin antibody [Santa Cruz Biotechnology, Dallas, TX, USA, #sc-8432]), all diluted 1:1000 in 3% BSA or NFDM prepared in TBS-T. After incubation with the primary antibody, the membranes were washed three times with TBS-T for 10 min each and then incubated for 1 h at RT with a secondary antibody against mouse or rabbit IgG, depending on the host of the primary antibody, coupled to HRP at a dilution of 1:3000. The membrane was washed three times with TBS-T for 10 min each, and protein bands were visualized using Pierce™ ECL Western Blotting Substrate (Thermo Fisher Scientific, Waltham, MA, USA, 32106). Finally, the relative levels of the target protein from 3 biological replicates were expressed in comparison to the control after normalization with a loading control or the total protein for phosphorylated targets.

### 2.8. Statistical Analysis

Data are presented as the mean  ±  S.D. of at least three independent experiments. In each graph, high-transparency dots indicate technical replicates, while white dots indicate biological replicates. Data was analyzed using Graphpad Prism 8.0 software, and the normality and S.D. of each dataset were evaluated before the statistical test was applied. In the case of Western blot and ATP level measurements, each condition was normalized to the DMSO condition within each independent experiment. Because all treatment conditions were measured within the same experiment, statistical analysis was performed using a one-way repeated-measures ANOVA. Multiple comparisons were conducted using Tukey’s post hoc test to compare all groups with each other. Statistical tests for other experiments and significance are indicated in each figure legend.

## 3. Results

### 3.1. c-Abl Localizes to Mitochondria in Neuronopathic GD RecNciI Mutant Fibroblasts

**Figure 1:**
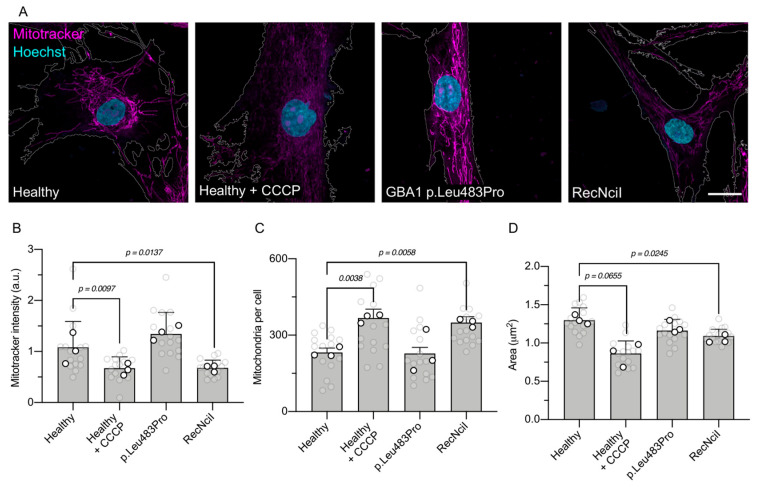
Mitochondrial alterations in fibroblasts derived from Gaucher patients. (**A**) Representative confocal images of fibroblasts from healthy donors treated with a vehicle or CCCP and fibroblasts from Gaucher patients carrying p.Leu483Pro or RecNciI mutations. Mitochondria were stained with Mitotracker (magenta), and nuclei were stained with DAPI (blue). Scale bar: 20 µm. (**B**) Quantification of Mitotracker fluorescence intensity per cell. (**C**) Quantification of the number of mitochondria per cell. (**D**) Quantification of mitochondrial area (µm^2^). Statistical analysis was performed using Welch’s ANOVA followed by Dunnett’s post hoc test. *p* values are indicated in each graph. (n = 3 biological replicates).

To evaluate mitochondrial status in fibroblasts from Gaucher patients, we first assessed mitochondrial homeostasis in fibroblasts with homozygous p.Leu483Pro and compound RecNciI (p.Leu483Pro, p.Ala495Pro, and p.Val499Val) mutations in the *GBA1* gene encoding for GCase. Mitochondrial membrane potential (∆Ψm) was measured using Mitotracker, a probe dependent on ∆Ψm, and its specificity was confirmed using CCCP, an oxidative phosphorylation uncoupler, which significantly reduced Mitotracker staining in healthy fibroblasts (Figure 1A,B).

We observed that fibroblasts expressing the homozygous p.Leu483Pro mutation did not exhibit a change in ∆Ψm compared to healthy fibroblasts. In contrast, RecNciI Gaucher fibroblasts displayed significantly reduced ∆Ψm (Figure 1A,B). Our analysis revealed that p.Leu483Pro fibroblasts did not show significant changes either in the number of mitochondria per cell or the size of mitochondria (Figure 1C,D), while RecNciI fibroblasts showed an increase in the number of mitochondria per cell and a reduction in mitochondrial size compared to healthy fibroblasts, consistent with mitochondrial fragmentation (Figure 1C,D). These changes were mirrored in healthy fibroblasts treated with CCCP, which exhibited increased mitochondrial numbers and decreased sizes (Figure 1C,D). Although both genotypes are associated with neuronopathic Gaucher disease, p.Leu483Pro typically presents with a later onset and a more slowly progressive neurological course, often within the Type 3 spectrum. In contrast, the RecNciI genotype, either in homozygosity or combined with another severe allele, is consistently linked to earlier onset and more aggressive neuronopathic disease, frequently manifesting as Type 2 GD or perinatal lethal phenotypes, with more severe neurological involvement than that observed in p.Leu483Pro patients [3,40]. These findings suggest that the compound RecNciI genotype may result in a more severe fragmented mitochondrial phenotype compared to p.Leu483Pro, with impaired compensation for mitochondrial alterations in RecNciI fibroblasts.

**Figure 2:**
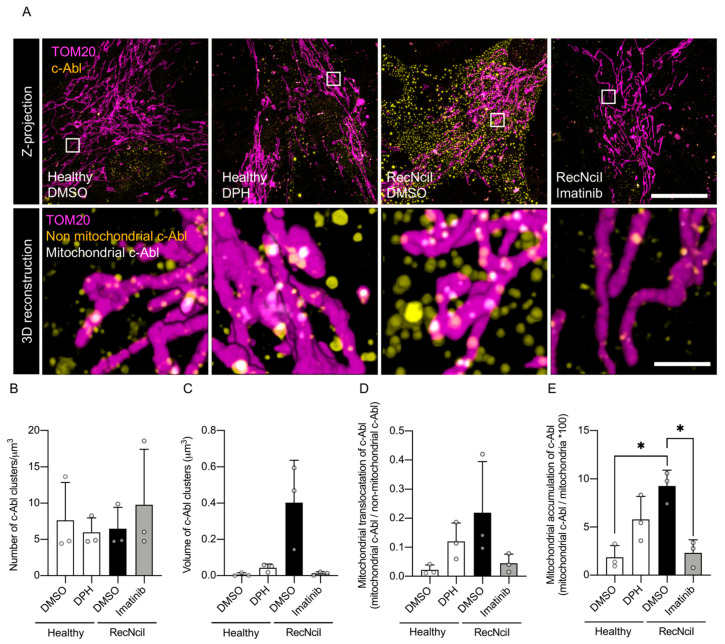
Activation-dependent mitochondrial localization of c-Abl drives mitochondrial dysfunction in RecNciI fibroblasts. (**A**) Z-projection (upper row) and 3D reconstructions of inset (lower row) from expansion microscopy showing healthy fibroblasts treated with a vehicle or DPH (c-Abl activator) and RecNciI fibroblasts treated with a vehicle or imatinib (c-Abl inhibitor). Samples were stained for c-Abl (yellow) and mitochondria (magenta); an overlapping signal corresponding to mitochondrial c-Abl is shown in white. Scale bar: 10 µm. Inset: 1 µm. (**B**) Quantification of the number of c-Abl clusters per µm^3^. (**C**) Quantification of c-Abl cluster volume (µm^3^). (**D**) Mitochondrial association of c-Abl ratio. (**E**) Percentage of mitochondrial accumulation of c-Abl. Statistical analysis was performed using the Kruskal–Wallis test followed by Dunn’s post hoc test; significant differences are indicated as * *p* < 0.05. (n = 3 biological replicates).

Importantly, the c-Abl tyrosine kinase has been shown to promote mitochondrial dysfunction following cellular damage [24,28,41]. Building on this, we previously reported that c-Abl is activated in GD models [19]. To further investigate whether c-Abl localizes to mitochondria in these cells, we employed expansion microscopy (ExM; Figure 2A), which enabled nanoscale visualization of c-Abl distribution relative to the mitochondrial marker TOM20.

First, we analyzed the nanoscale organization of c-Abl clusters under different conditions. DPH treatment of healthy fibroblasts, which pharmacologically activate c-Abl, did not change the number of c-Abl clusters but produced a mild, non-significant increase in cluster volume (Figure 2B,C). Similarly, RecNciI fibroblasts showed no significant change in cluster numbers but exhibited a tendency toward larger cluster volumes compared to control fibroblasts. Because larger clusters are visually easier to detect than smaller ones, this increase in cluster size may contribute to an apparent increase in cluster density, although quantitative analysis indicated no changes in cluster number but rather an increase in cluster size. Treatment of RecNciI fibroblasts with the c-Abl inhibitor imatinib did not alter cluster number but reduced cluster volume toward control levels, consistent with a role of imatinib in modulating c-Abl activity (Figure 2B,C).

To test whether c-Abl activation affected its mitochondrial localization, we quantified both the mitochondrial association and the mitochondrial accumulation of c-Abl (Figure 2D,E). The c-Abl association was evaluated by calculating the ratio of mitochondrial to non-mitochondrial c-Abl, indicating the enrichment of c-Abl within mitochondrial regions compared to the rest of the cell (Figure 2D). In parallel, mitochondrial accumulation was assessed by determining the percentage of TOM20-defined mitochondrial volume occupied by c-Abl, providing a measure of how extensively c-Abl associates with and covers the mitochondrial network (Figure 2E). DPH-treated healthy fibroblasts displayed a tendency towards an increase in both mitochondrial association and accumulation of c-Abl, suggesting that pharmacological activation of c-Abl could promote its recruitment and enrichment at the mitochondria. In line with this, RecNciI fibroblasts also exhibited elevated mitochondrial c-Abl levels (Figure 2D,E). Remarkably, imatinib treatment normalized these parameters, reducing both mitochondrial association and accumulation to levels comparable to healthy fibroblasts. These findings suggest a direct link between c-Abl activity and its mitochondrial localization, in which pharmacological or pathological c-Abl activation is both sufficient and necessary to promote its association with the mitochondria.

### 3.2. c-Abl Inhibition Reduces RIPK3 Activation in CBE-Treated Cortical Neurons

**Figure 3:**
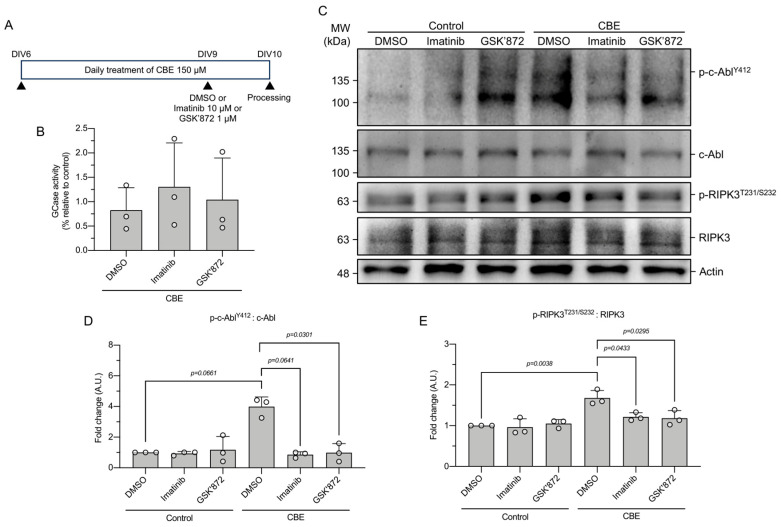
c-Abl inhibition restores mitochondrial function in CBE-treated primary cortical neurons. (**A**) Schematic representation of the experimental design for CBE treatment in primary cortical neurons. (**B**) Measurement of GCase activity following CBE treatment. (**C**) Representative immunoblots of p-c-Abl^Y412^, c-Abl, p-RIPK3^T231/S232^, RIPK3 and actin from cortical neurons. (**D**) Quantification of the ratio of p-c-Abl^Y412^:c-Abl. (**E**) Quantification of the ratio of p-RIPK3^T231/S232^:RIPK3. Statistical analysis was performed using repeated-measures ANOVA followed by Bonferroni’s post hoc test; significant differences are indicated (n = 3 biological replicates).

We have previously reported that activation of c-Abl is associated with increased RIPK3 activation in RecNciI fibroblasts [19]. While fibroblasts showed some evidence of how c-Abl impacts mitochondrial homeostasis in cells from a neuronopathic Gaucher patient, such findings are limited to a model due to their non-neuronal nature and inability to fully recapitulate the disease-relevant neuronal context. To determine whether a similar relationship occurs in a pharmacological neuronal model of neuronopathic GD, cortical neurons were treated with CBE, an irreversible inhibitor of GCase, from day 6 to day 10 (Figure 3A). Measurement of GCase enzymatic activity showed that CBE treatment significantly decreased activity versus control neurons, and this was independent of the presence of imatinib or GSK’872, confirming the effectiveness of the pharmacological model. (Figure 3B).

At DIV9, either 10 μM imatinib or 1 μM of the RIPK3 inhibitor GSK’872 was added to the cells to determine the activation status of c-Abl and RIPK3 by assessing their phosphorylated forms. Immunoblot analysis revealed that CBE treatment led to a significant increase in phosphorylated c-Abl at Y412 (p-c-Abl^Y412^) and phosphorylated RIPK3 at T231 and S232 (p-RIPK3^T231/S232^) compared to control neurons, indicating enhanced kinase activation under conditions of chronic GCase inhibition (Figure 3C–E).

Importantly, treatment of CBE-exposed neurons with either imatinib or GSK’872 resulted in a significant reduction in both p-c-Abl^Y412^ and p-RIPK3^T231/S232^ levels relative to CBE alone (Figure 3C–E). The ability of imatinib, a c-Abl inhibitor, to reduce RIPK3 phosphorylation, as well as the reciprocal effect observed with GSK’872 on c-Abl phosphorylation, suggests the existence of functional crosstalk between c-Abl and RIPK3 signaling pathways in CBE-treated cortical neurons. Together, these results indicate that chronic pharmacological inhibition of GCase leads to concomitant activation of c-Abl and RIPK3 in cortical neurons and that inhibition of either kinase attenuates the activation of both, suggesting a model in which c-Abl and RIPK3 signaling are functionally linked in neuronal responses to GCase dysfunction.

### 3.3. c-Abl or RIPK3 Inhibition Improves Mitochondrial Function in a Neuronopathic Gaucher Model

**Figure 4:**
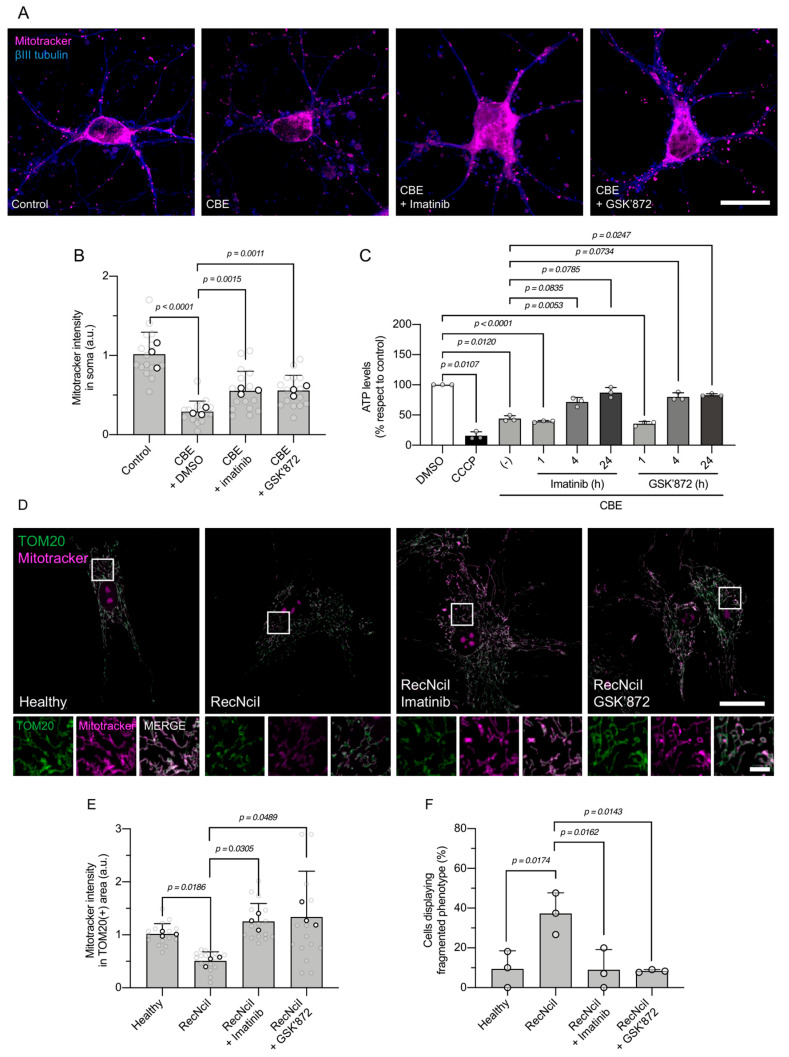
Pharmacological inhibition of c-Abl or RIPK3 restores mitochondrial function and morphology in neuronal and genetic models of Gaucher disease. (**A**) Representative confocal images of cortical neurons stained for Mitotracker (magenta) and βIII tubulin (blue). Scale bar: 20 μm. (**B**) Quantification of MitoTracker fluorescence intensity in neuronal somas from neurons. (**C**) Intracellular ATP levels measured in neurons treated with CBE or CCCP or with imatinib or GSK’872, at the indicated times. (**D**) Representative confocal images of fibroblasts stained for TOM20 (green) and MitoTracker (magenta). The white box indicates the region shown at higher magnification in the lower row. (**E**) Quantification of MitoTracker fluorescence intensity within the TOM20-positive area. Scale bar: 20 μm. Scale bar of inset: 2 μm. (**F**) Quantification of mitochondrial morphology showing the proportion of cells with a fragmented mitochondrial network. Data are presented as mean ± SD. Statistical analysis was performed using Welch’s ANOVA, followed by Dunnett’s post hoc test for (**B**,**E**,**F**) and repeated-measure ANOVAs followed by Dunn’s post hoc test for (**C**); significant differences are indicated (n = 3 biological replicates).

Since active c-Abl is correlated to increased mitochondria and the c-Abl-RIPK3 axis is altered in both genetic and pharmacological models of GD, we asked whether inhibition of c-Abl and RIPK3 has an impact on mitochondrial status. Importantly, CBE-treated neurons displayed a significant reduction in Mitotracker intensity in neuronal somas (Figure 4A,B), consistent with what was seen in RecNciI fibroblasts, while pharmacological inhibition of c-Abl or RIPK3 led to a significant increase in Mitotracker intensity in neuronal somas of CBE-treated neurons (Figure 4A,B).

To further assess mitochondrial bioenergetics in the pharmacological model, we measured ATP levels in CBE-treated neurons. CBE treatment led to a significant reduction in ATP levels, indicating impaired ATP production, as well as CCCP treatment (Figure 4C). Treatment with imatinib or GSK’872 restored ATP levels in a time-dependent manner, with significant increases observed after 4 and 24 h of treatment (Figure 4C).

To determine whether this phenotype was conserved in the genetic model of GD, we next examined mitochondrial status in RecNciI fibroblasts. Cells were co-stained with TOM20 to label total mitochondrial mass independently of ΔΨm, and MitoTracker to assess functional mitochondria (Figure 4D). Consistent with our previous observations (Figure 1A,B), MitoTracker intensity within the TOM20-positive area was significantly reduced in RecNciI compared to healthy fibroblasts (Figure 4D,E). Importantly, pharmacological inhibition of either c-Abl with imatinib or RIPK3 with GSK’872 significantly increased MitoTracker intensity in the TOM20-positive area, indicating a restoration of mitochondrial functional status (Figure 4D,E).

Because TOM20 staining allows visualization of the total mitochondrial network regardless of ΔΨm, we further classified mitochondrial morphology based on network integrity. RecNciI fibroblasts displayed a significantly higher proportion of cells with a fragmented mitochondrial phenotype compared to healthy fibroblasts (Figure 4F). Notably, treatment with either imatinib or GSK’872 markedly reduced the prevalence of this fragmented phenotype, promoting a more interconnected mitochondrial network (Figure 4F).

Together, these data demonstrate that both pharmacological and genetic models of GD exhibit impaired mitochondrial function and altered mitochondrial morphology, and that inhibition of the c-Abl–RIPK3 axis improves mitochondrial status across neuronal and non-neuronal cell types.

### 3.4. Inhibition of c-Abl Promotes Autophagic Engulfment of Mitochondria in Gaucher Disease Models

A key mechanism for maintaining mitochondrial homeostasis is the selective removal of dysfunctional mitochondria through autophagy, known as mitophagy, in which dysfunctional mitochondria are engulfed by LC3-rich autophagosomes to be delivered to lysosomes for their degradation. To determine whether dysregulation of the c-Abl–RIPK3 axis affects autophagy and mitophagy in GD, we first assessed autophagic flux in cortical neurons using biochemical markers.

**Figure 5:**
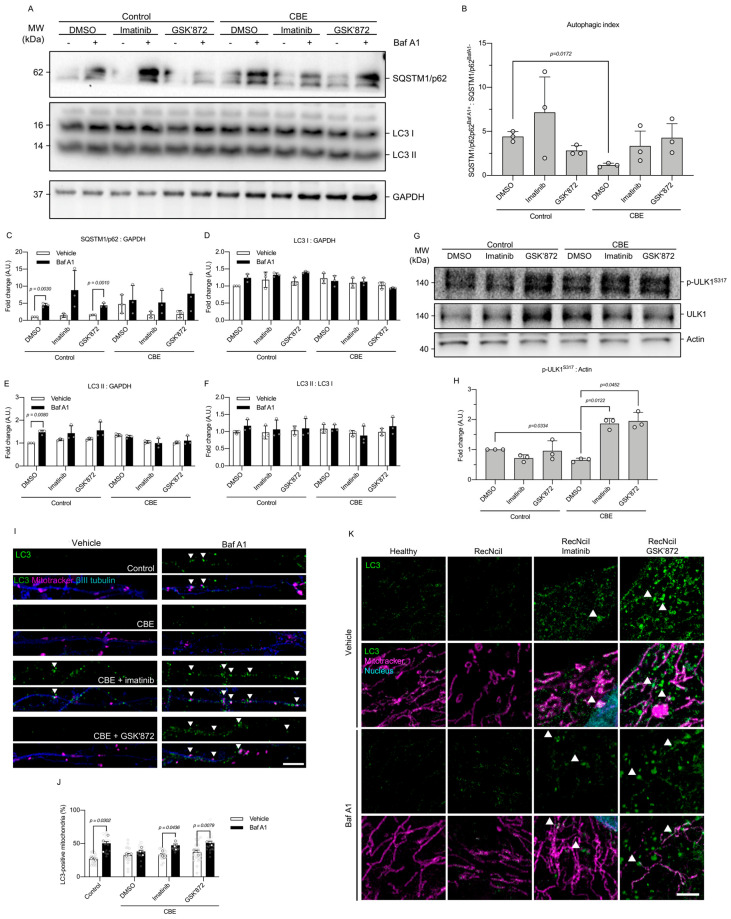
Inhibition of the c-Abl–RIPK3 axis restores autophagy initiation and mitophagy in neuronal and fibroblast models of Gaucher disease. (**A**) Representative immunoblots of p62/SQSTM1 and LC3 in cortical neurons under control and CBE-treated conditions, in the presence (+) or absence (−) of Bafilomycin A1 (Baf A1), and following treatment with imatinib or GSK’872. (**B**) Autophagic index expressed as the ratio of p62/SQSTM1 levels in Baf A1-treated versus untreated conditions. (**C**) Quantification of p62/SQSTM1 protein levels. (**D**–**F**) Quantification of LC3-I (**D**), LC3-II (**E**), and LC3-II/LC3-I ratio (**F**) normalized to GAPDH. (**G**) Representative immunoblots of phosphorylated ULK1 at Ser317 (p-ULK1^S317^) and total ULK1 in cortical neurons. (**H**) Quantification of p-ULK1^S317^ levels normalized to actin. (**I**) Representative confocal images of proximal neurites from cortical neurons stained for LC3 (green), MitoTracker (magenta), and βIII-tubulin (blue). Arrowheads indicate LC3-positive mitochondria. (**J**) Quantification of LC3-positive mitochondria. (**K**) Representative confocal images of fibroblasts stained for LC3 (green), MitoTracker (magenta), and nuclei (blue). Arrowheads indicate mitochondria positive for LC3. Scale bar: 2 μm. Data are presented as mean ± SD. Statistical analysis was performed using Welch’s ANOVA, followed by Dunnett’s post hoc test for (**B**,**J**) and repeated-measures ANOVA followed by Dunn’s post hoc test for (**C**–**F**,**H**); significant differences are indicated (n = 3 biological replicates).

Immunoblot analysis of p62/SQSTM1 and LC3 in cortical neurons revealed a significant reduction in the autophagic index in CBE-treated neurons compared to control conditions (Figure 5A,B), indicating an impaired autophagic flux. Consistent with this, p62/SQSTM1 levels accumulated in control neurons upon Bafilomycin A1 (Baf A1) treatment, whereas CBE -treated neurons showed elevated basal p62/SQSTM1 levels and failed to further accumulate p62 in response to Baf A1 (Figure 5C), suggesting a block in the autophagic flux. Importantly, pharmacological inhibition of c-Abl or RIPK3 in CBE-treated neurons restored autophagic flux, as assessed by p62/SQSTM1 turnover, reducing p62 levels to values not significantly different from control neurons, despite no detectable changes in LC3-I, LC3-II, or LC3-II/LC3-I ratios found using Western blot. Accordingly, LC3-I, LC3-II, and LC3-II/LC3-I ratios remained unchanged across conditions, except for a modest increase in LC3-II in control neurons treated with Baf A1 (Figure 5D–F).

Given that initiation of autophagy is regulated by serine/threonine (S/T) kinase ULK1 activation, we next examined ULK1 phosphorylation at S317 (p-ULK1^S317^), which is associated with increased activity of this S/T kinase. Interestingly, CBE treatment significantly reduced p-ULK1^S317^ levels compared to control neurons (Figure 5G,H), indicating impaired activation of the autophagy initiation machinery. Importantly, pharmacological inhibition of either c-Abl (imatinib) or RIPK3 (GSK’872) significantly increased ULK1 phosphorylation in CBE-treated neurons (Figure 5H), suggesting that the c-Abl–RIPK3 axis negatively regulates autophagy initiation in this model.

To determine whether an improvement in autophagy translated into enhanced mitophagy, we examined LC3–mitochondria colocalization using confocal microscopy. In proximal neurites, control neurons displayed increased numbers of LC3-positive mitochondria following Baf A1 treatment, indicative of ongoing mitophagy (Figure 5I,J). In contrast, CBE-treated neurons failed to show LC3 accumulation on mitochondria, consistent with defective autophagosome formation. Notably, treatment with imatinib or GSK’872 significantly increased the number of LC3-positive mitochondria in CBE-treated neurons in the presence of Baf A1 (Figure 5I,J), indicating restored mitophagic engulfment. A similar phenotype was observed in the genetic GD model. In RecNciI fibroblasts, LC3–mitochondria colocalization was similarly compared to healthy fibroblasts; however, these cells showed an impaired mitochondria phenotype (Figure 1A,B and Figure 4D,F). By contrast, treatment with imatinib or GSK’872 significantly increased the proportion of mitochondria positive for LC3 (Figure 5K), suggesting that inhibition of the c-Abl–RIPK3 axis promotes mitophagy in both pharmacological and genetic models of GD.

Together, these data indicate that GD is associated with defective autophagy initiation and impaired mitophagy, driven in part by aberrant c-Abl–RIPK3 signaling. Pharmacological inhibition of this pathway restores ULK1 activation, autophagosome formation, and mitochondrial engulfment, supporting a mechanistic link between c-Abl activity and mitochondrial quality control in GD.

## 4. Discussion

The clinical management of GD has significantly improved with enzyme replacement therapy and substrate reduction therapy. However, for neuronopathic forms, such as Type 2 and Type 3, therapeutic efficacy remains limited [6]. Here, we address mitochondrial impairment with altered c-Abl/RIPK3 signaling in mutant fibroblasts from patients displaying neurological impairment and in a neuronal model using an inhibitor of GCase.

Previous studies have described alterations in mitochondrial status in various *GBA1*-associated PD and GD models. By using Mitotracker staining to visualize the mitochondrial network, we observed distinct ∆Ψm profiles in different *GBA1* mutant fibroblasts from neuronopathic Gaucher patients. *GBA1* p.Leu483Pro fibroblasts exhibited no change in ∆Ψm levels compared to healthy fibroblasts, whereas *GBA1* RecNciI fibroblasts carrying the heterozygous mutations p.Ala495Pro and p.Val499Val alongside the homozygous p.Leu483Pro mutation in GCase, displayed significantly lower ∆Ψm levels.

Further examination of mitochondrial health revealed differential alterations, with mitochondria from GCase RecNciI fibroblasts displaying a more fragmented phenotype than p.Leu483Pro fibroblasts, associated with an increased number of smaller mitochondria. Despite the severe phenotype associated with the p.Leu483Pro mutation in GD Types 2 and 3, a combination of p.Leu483Pro and RecNciI results in Type 2 GD with more severe neurological involvement. Importantly, recent findings have revealed that both WT and mutant GCase exhibit not only lysosomal functionality but also the ability to translocate to mitochondria. Notably, WT GCase has been shown to exert a stabilizing effect on complex I of the OXPHOS system in HEK-293 cells and in iPSC-derived dopaminergic neurons [42]. In contrast, disease-associated mutations in GCase compromise the stability and function of complex I while enhancing interactions with and dysregulating the mitochondrial quality control machinery [42]. Thus, complex alleles associated with increased severity of the GD phenotype present more profound mitochondrial alterations, potentially leading to cellular malfunctions incapable of compensating for mitochondrial changes.

In the neuronal pharmacological model, CBE treatment led to a mitochondrial phenotype similar to that of GCase RecNciI fibroblasts, including a decrease in ∆Ψm. Maintenance of ΔΨm by the electron transport chain is essential for oxidative phosphorylation and ATP synthesis. Previously, it has been shown that in CBE-treated SH-SY5Y cells, a neuroblastoma cell line, the phosphorylation of ADP is significantly inhibited [9]. This inhibition is in line with an increase in oxidative stress and fragmentation of mitochondria. Consistent with this, our results demonstrated a decrease in ATP levels when neurons were exposed to CBE, suggesting that CBE treatment in primary cortical neurons reproduces major mitochondrial alterations associated with a more severe neuronopathic Gaucher phenotype. In addition, GCase deficiency has been shown to alter mitochondria–lysosome contact dynamics. Prolonged mitochondria–lysosome tethering was reported in human dopaminergic neurons following CBE treatment, suggesting the defective untethering of contact. Such persistent contacts may facilitate aberrant transfer of lipids or Ca^2+^ and interfere with mitochondrial dynamics, thereby propagating lysosomal dysfunction to mitochondria [43,44]. Given that mitochondrial dysfunction is a major source of ROS, this redox imbalance may further amplify neuronal injury in GD [13,14,15]. Indeed, although mitochondrial dysfunction is a well-recognized feature of GD, the upstream signaling events driving or maintaining these alterations remain poorly understood. Therefore, therapeutic strategies that restore or enhance mitophagy by modulating key signaling pathways and upstream regulators of mitochondrial quality control are critical.

Importantly, oxidative stress is increasingly recognized as a central component of GD. Multiple studies support the presence of redox imbalance in GD models and patients, and it has been shown that *GBA1* deficiency increases intracellular ROS in cultured fibroblasts [6]. Clinically, GD patients exhibit elevated oxidative stress markers and reduced antioxidant capacity. Antioxidant-based approaches have, therefore, been explored [6]. Approaches such as vitamin E supplementation have been proposed since reduced vitamin E and increased lipid peroxidation have been reported in GD patients [45]. Additionally, among the currently promising options for GD Type 2, ambroxol stands out as a pharmacological chaperone that penetrates the CNS at high doses [6,46,47]. Indeed, case reports show improvement or containment of neuroinflammation and some biomarkers associated with cellular stress [47,48].

In this work, we show that the c-Abl-RIPK3 signaling axis contributes to mitochondrial dysfunction and that its inhibition promotes mitophagy, thereby interrupting downstream pathways of cellular damage. Specifically, we observed that both pharmacological activation of the stress-responsive c-Abl tyrosine kinase in healthy fibroblasts and pathological activation of c-Abl in RecNciI fibroblasts derived from a Gaucher patient displayed increased levels of c-Abl within the mitochondria. This accumulation was assessed using ExM, a powerful imaging approach that offers nanoscale resolution while preserving spatial relationships within cellular structures. Indeed, ExM allowed us to precisely localize c-Abl in proximity to mitochondrial markers, providing direct visual evidence of its mitochondrial association under pathological or pharmacological conditions. These findings are in line with our recent report demonstrating that c-Abl translocates to the mitochondria in response to endoplasmic reticulum stress [28]. This observation also agrees with earlier studies indicating that c-Abl can localize to mitochondria under stress conditions [25,26], and that other cytosolic kinases, including ERK1/2, undergo similar translocation events that regulate processes such as mitophagy [49]. Importantly, we show that pharmacological inhibition of c-Abl leads to a reduction in its mitochondrial association, further supporting the dynamic regulation of its subcellular localization. Together, these findings suggest that mitochondrial association of c-Abl may represent a conserved stress response mechanism, potentially contributing to the dysregulation of mitochondrial quality control pathways in GD.

Our laboratory previously described the activation of the c-Abl-RIPK3 signaling pathway in GD, observing elevated levels of phosphorylated c-Abl and RIPK3 in different Gaucher models [19]. Interestingly, here, we found that inhibition of these kinases with imatinib and GSK’872 treatments restored mitochondrial function in Gaucher RecNciI fibroblasts and CBE-treated neurons. Notably, both c-Abl and RIPK3 kinases can localize to the mitochondria and induce their fragmentation and dysfunction in the context of neurodegeneration. For instance, c-Abl has been shown to directly phosphorylate Drp1, a protein involved in mitochondrial fission, promoting mitochondrial fragmentation and contributing to oxidative-stress-induced cell death in cortical neurons [24]. Importantly, Drp1 deficiency leads to RIPK3-mediated neurodegeneration [50], indicating a complex crosstalk between c-Abl-RIPK3 signaling and mitochondrial function. In line with this, and the damage caused by ischemia and reperfusion in renal and cardiac tissues, RIPK3 has been described to translocate to the mitochondria, triggering mitochondrial damage that contributes to cellular and tissue deterioration [51]. Necroptosis inhibition also protects against axonal degeneration mediated by mitochondrial fragmentation and dysfunction [52]. Similarly, c-Abl activation has been observed in amyotrophic lateral sclerosis (ALS) models, phosphorylating the mitochondrial fusion protein, MFN2, leading to mitochondrial fragmentation and dysfunction mediated by oxidative stress, resulting in motor neuron death [28]. Thus, our results suggest that c-Abl and RIPK3 negatively regulate mitochondrial function in GD. In this context, since both kinases can localize to the mitochondria, it would be of particular interest to determine whether c-Abl and RIPK3 physically or functionally interact at the mitochondria, either at the mitochondrial membranes or within mitochondrial compartments.

Autophagy, a crucial cellular process regulating mitochondrial homeostasis, is dysregulated in GD. Our results showed that CBE treatment disrupted autophagic flux in cortical neurons, indicated by increased SQSTM1/p62 levels and impaired autophagosome formation, together with impairment of mitophagy, by disrupting the sequestration of mitochondria within autophagosomes, an event that was also observed in Gaucher RecNciI fibroblasts, indicating a failure to recognize and target dysfunctional mitochondria for degradation. Importantly, c-Abl activation is implicated in the dysregulation of autophagy, and its inhibition promotes cell survival by modulating autophagy in various neurodegenerative diseases such as PD and other LSDs [53,54,55,56].

In neuronal models, inhibition of c-Abl with the tyrosine kinase inhibitor nilotinib reduces mTORC1 activity while increasing AMPK phosphorylation and ULK1 activation, thereby enhancing autophagic flux [55]. Consistent with these observations, c-Abl activity has also been linked to parkin-dependent ubiquitination of mitochondrial proteins and mitochondrial quality control mechanisms associated with neurodegeneration [57,58], supporting a model in which c-Abl negatively regulates early autophagy signaling upstream of AMPK–ULK1 and mitophagy pathways. Notably, the influence of c-Abl on mTORC1 appears to be context-dependent. While Karim et al. [55] reported mTORC1 down-regulation upon c-Abl inhibition in neuronal cell lines, previous work from our laboratory showed that c-Abl inhibition does not alter mTORC1 activation in HeLa cells [56]. These differences suggest that c-Abl intersects with AMPK–mTORC1 signaling in a cell-type and metabolic-state-dependent manner. Additionally, RIPK3 has been associated with different effects on autophagy, and previous studies have uncovered a crosstalk between RIPK3 and the PINK1/Parkin pathway, modulated by AMPK [31,36,59]. Moreover, beyond the PINK1/Parkin pathway, mitophagy receptors may also mediate the inability to induce mitophagy in neuronopathic GD. Previous studies have demonstrated interactions between RIPK3 and mitophagy receptors such as FUNDC1 or BNIP3 [30,51,60], resulting in dysregulated mitophagy. However, the implications of these interactions in a neurodegenerative context need further investigation; therefore, it is reasonable to expect that inhibition of c-Abl or RIPK3 could restore the autophagy process in the GD models used.

Indeed, our experimental observations revealed impaired ULK1 activity in neurons treated with CBE, indicated by the absence of change in p-ULK1^S317^ levels compared to the control. Notably, treatment with imatinib and GSK’872 significantly increased p-ULK1^S317^ levels without affecting basal levels. p-ULK1^S317^ is a key molecular event associated with the induction of autophagy. This residue is directly phosphorylated by AMPK under conditions of energetic or metabolic stress, promoting ULK1 activation and initiation of the autophagy program. Phosphorylation at S317 facilitates the assembly and activation of the ULK1 complex, thereby triggering downstream recruitment of autophagy machinery required for phagophore formation [61,62,63,64,65]. Accordingly, reduced ULK1^S317^ phosphorylation is commonly associated with impaired autophagy initiation, whereas increased phosphorylation at this site reflects activation of upstream autophagic signaling and enhanced autophagosome biogenesis. Thus, consistent with prior reports [55], our results imply a negative regulatory role of c-Abl and RIPK3 in inducing autophagy.

In line with this, imatinib and GSK’872 treatments alleviated these impairments, together with an improvement in autophagic engulfment of mitochondria, supporting the idea that c-Abl and RIPK3 are involved in early-stage autophagy impairment in GD. Over time, this failure to remove damaged mitochondria can sensitize cells to stress and contribute to progressive cellular dysfunction, particularly in energetically demanding cell types such as neurons, and targeting the signaling contributing to this impairment could potentially restore the ability of cells to identify and degrade dysfunctional mitochondria to restore cellular homeostasis in this condition.

The results of this study, together with previous findings [19], indicate that c-Abl is a promising therapeutic target for GD Type 2 since its inhibition blocks signaling pathways involving RIPK3 and mitochondrial dysfunction. This supports the use of blood–brain barrier-penetrant c-Abl inhibitors such as Neurotinib, which have been shown to reduce neuronal damage in multiple preclinical models of brain disease, including Niemann-Pick A [54], Alzheimer’s disease [66], and epilepsy [67].

## 5. Conclusions

In neuronopathic Gaucher disease, *GBA1* mutations trigger lysosomal dysfunction and cellular stress responses that may initiate aberrant signaling cascades. Our findings support a model in which GCase deficiency promotes the activation and mitochondrial association of the cytoplasmic tyrosine kinase c-Abl. Once localized to mitochondria, c-Abl may contribute to the recruitment or activation of RIPK3, a kinase previously associated with mitochondrial stress responses and regulated forms of cell death. The engagement of this c-Abl–RIPK3 axis at the mitochondria appears to interfere with mitophagy, as evidenced by reduced mitochondrial engulfment and altered autophagic flux. As a result, dysfunctional mitochondria accumulate, exacerbating mitochondrial stress and likely promoting increased oxidative stress, contributing to neuronal vulnerability in neuronopathic GD. Given that mitochondrial dysfunction is a major source of ROS, this redox imbalance may further promote cellular damage in affected neurons. Future studies should delineate the molecular interactions among c-Abl, RIPK3, and autophagic machinery in the context of mitochondrial dysfunction and evaluate the translational potential of their pharmacological inhibition in preclinical Gaucher models, including patient-derived neuronal and mouse models.

## Data Availability

All data supporting the findings of this study are available within the article and its figures. No additional datasets were generated or analyzed during the current study.

## References

[1] Huang W.J., Zhang X., Chen W.W. (2015). Gaucher Disease: A Lysosomal Neurodegenerative Disorder. Eur. Rev. Med. Pharmacol. Sci..

[2] Arévalo N.B., Lamaizon C.M.M., Cavieres V.A., Burgos P.V., Álvarez A.R., Yañez M.J., Zanlungo S. (2022). Neuronopathic Gaucher Disease: Beyond Lysosomal Dysfunction. Front. Mol. Neurosci..

[3] Hruska K.S., LaMarca M.E., Scott C.R., Sidransky E. (2008). Gaucher Disease: Mutation and Polymorphism Spectrum in the Glucocerebrosidase Gene (GBA). Hum. Mutat..

[4] Grabowski G.A. (2008). Phenotype, Diagnosis, and Treatment of Gaucher’s Disease. Lancet.

[5] Riboldi G.M., Di Fonzo A.B. (2019). GBA, Gaucher Disease, and Parkinson’s Disease: From Genetic to Clinic to New Therapeutic Approaches. Cells.

[6] Dewsbury M., Purcell T., Hughes D., Donald A., Hargreaves I.P., Stepien K.M. (2025). Secondary Mitochondrial Dysfunction in Gaucher Disease Type I, II and III—Review of the Experimental and Clinical Evidence. Genes.

[7] Osellame L.D., Rahim A.A., Hargreaves I.P., Gegg M.E., Richard-Londt A., Brandner S., Waddington S.N., Schapira A.H.V., Duchen M.R. (2013). Mitochondria and Quality Control Defects in a Mouse Model of Gaucher Disease—Links to Parkinson’s Disease. Cell Metab..

[8] Ivanova M.M., Changsila E., Iaonou C., Goker-Alpan O. (2019). Impaired Autophagic and Mitochondrial Functions Are Partially Restored by ERT in Gaucher and Fabry Diseases. PLoS ONE.

[9] Cleeter M.W.J., Chau K.-Y., Gluck C., Mehta A., Hughes D.A., Duchen M., Wood N.W., Hardy J., Mark Cooper J., Schapira A.H. (2013). Glucocerebrosidase Inhibition Causes Mitochondrial Dysfunction and Free Radical Damage. Neurochem. Int..

[10] Gegg M.E., Schapira A.H.V. (2016). Mitochondrial Dysfunction Associated with Glucocerebrosidase Deficiency. Neurobiol. Dis..

[11] Li H., Ham A., Ma T.C., Kuo S.H., Kanter E., Kim D., Ko H.S., Quan Y., Sardi S.P., Li A. (2019). Mitochondrial Dysfunction and Mitophagy Defect Triggered by Heterozygous GBA Mutations. Autophagy.

[12] Nicholls D.G., Budd S.L. (2000). Mitochondria and Neuronal Survival. Physiol. Rev..

[13] Deganuto M., Pittis M.G., Pines A., Dominissini S., Kelley M.R., Garcia R., Quadrifoglio F., Bembi B., Tell G. (2007). Altered Intracellular Redox Status in Gaucher Disease Fibroblasts and Impairment of Adaptive Response against Oxidative Stress. J. Cell. Physiol..

[14] Kartha R.V., Terluk M.R., Brown R., Travis A., Mishra U.R., Rudser K., Lau H., Jarnes J.R., Cloyd J.C., Weinreb N.J. (2020). Patients with Gaucher Disease Display Systemic Oxidative Stress Dependent on Therapy Status. Mol. Genet. Metab. Rep..

[15] de la Mata M., Cotán D., Oropesa-Ávila M., Garrido-Maraver J., Cordero M.D., Villanueva Paz M., Delgado Pavón A., Alcocer-Gómez E., de Lavera I., Ybot-González P. (2015). Pharmacological Chaperones and Coenzyme Q10 Treatment Improves Mutant β-Glucocerebrosidase Activity and Mitochondrial Function in Neuronopathic Forms of Gaucher Disease. Sci. Rep..

[16] Ding W.-X., Yin X.-M. (2012). Mitophagy: Mechanisms, Pathophysiological Roles, and Analysis. Biol. Chem..

[17] Chu C. (2019). Mechanisms of Selective Autophagy and Mitophagy: Implications for Neurodegenerative Diseases. Neurobiol. Dis..

[18] Corti O. (2019). Neuronal Mitophagy: Lessons from a Pathway Linked to Parkinson’s Disease. Neurotox. Res..

[19] Yañez M.J., Campos F., Marín T., Klein A.D., Futerman A.H., Alvarez A.R., Zanlungo S. (2021). C-Abl Activates RIPK3 Signaling in Gaucher Disease. Biochim. Biophys. Acta Mol. Basis Dis..

[20] Gutiérrez D., Chandía-Cristi A., Yáñez M., Zanlungo S., Álvarez A. (2023). C-Abl Kinase at the Crossroads of Healthy Synaptic Remodeling and Synaptic Dysfunction in Neurodegenerative Diseases. Neural Regen. Res..

[21] Kharbanda S., Ren R., Pandey P., Shafman T.D., Feller S.M., Weichselbaum R.R., Kufe D.W. (1995). Activation of the C-Abl Tyrosine Kinase in the Stress Response to DNA-Damaging Agents. Nature.

[22] Sun X., Majumder P., Shioya H., Wu F., Kumar S., Weichselbaum R., Kharbanda S., Kufe D. (2000). Activation of the Cytoplasmic C-Abl Tyrosine Kinase by Reactive Oxygen Species. J. Biol. Chem..

[23] Klein A., Maldonado C., Vargas L.M., Gonzalez M., Robledo F., Perez de Arce K., Muñoz F.J., Hetz C., Alvarez A.R., Zanlungo S. (2011). Oxidative Stress Activates the C-Abl/P73 Proapoptotic Pathway in Niemann-Pick Type C Neurons. Neurobiol. Dis..

[24] Zhou L., Zhang Q., Zhang P., Sun L., Peng C., Yuan Z., Cheng J. (2017). C-Abl-Mediated Drp1 Phosphorylation Promotes Oxidative Stress-Induced Mitochondrial Fragmentation and Neuronal Cell Death. Cell Death Dis..

[25] Ito Y., Pandey P., Mishra N., Kumar S., Narula N., Kharbanda S., Saxena S., Kufe D. (2001). Targeting of the C-Abl Tyrosine Kinase to Mitochondria in Endoplasmic Reticulum Stress-Induced Apoptosis. Mol. Cell. Biol..

[26] Kumar S., Bharti A., Mishra N.C., Raina D., Kharbanda S., Saxena S., Kufe D. (2001). Targeting of the C-Abl Tyrosine Kinase to Mitochondria in the Necrotic Cell Death Response to Oxidative Stress. J. Biol. Chem..

[27] Katsumata R., Ishigaki S., Katsuno M., Kawai K., Sone J., Huang Z., Adachi H., Tanaka F., Urano F., Sobue G. (2012). C-Abl Inhibition Delays Motor Neuron Degeneration in the G93A Mouse, an Animal Model of Amyotrophic Lateral Sclerosis. PLoS ONE.

[28] Martinez A., Lamaizon C.M., Valls C., Llambi F., Leal N., Fitzgerald P., Guy C., Kamiński M.M., Inestrosa N.C., van Zundert B. (2023). C-Abl Phosphorylates MFN2 to Regulate Mitochondrial Morphology in Cells under Endoplasmic Reticulum and Oxidative Stress, Impacting Cell Survival and Neurodegeneration. Antioxidants.

[29] Vitner E.B., Salomon R., Farfel-Becker T., Meshcheriakova A., Ali M., Klein A.D., Platt F.M., Cox T.M., Futerman A.H. (2014). RIPK3 as a Potential Therapeutic Target for Gaucher’s Disease. Nat. Med..

[30] Luo B., Ming Z. (2023). Uncovering Interactions of Mitochondrial Proteins BNIP3 and BNIP3L with Necroptosis-Associated Kinase RIPK3: Insights into Kinase Activation. Biochem. Biophys. Res. Commun..

[31] Wu W., Wang X., Sun Y., Berleth N., Deitersen J., Schlütermann D., Stuhldreier F., Wallot-Hieke N., José Mendiburo M., Cox J. (2021). TNF-Induced Necroptosis Initiates Early Autophagy Events via RIPK3-Dependent AMPK Activation, but Inhibits Late Autophagy. Autophagy.

[32] Matsuzawa-Ishimoto Y., Shono Y., Gomez L.E., Hubbard-Lucey V.M., Cammer M., Neil J., Dewan M.Z., Lieberman S.R., Lazrak A., Marinis J.M. (2017). Autophagy Protein ATG16L1 Prevents Necroptosis in the Intestinal Epithelium. J. Exp. Med..

[33] Goodall M.L., Fitzwalter B.E., Zahedi S., Wu M., Rodriguez D., Mulcahy-Levy J.M., Green D.R., Morgan M., Cramer S.D., Thorburn A. (2016). The Autophagy Machinery Controls Cell Death Switching between Apoptosis and Necroptosis. Dev. Cell.

[34] Lee S.B., Kim J.J., Han S.-A., Fan Y., Guo L.-S., Aziz K., Nowsheen S., Kim S.S., Park S.-Y., Luo Q. (2019). The AMPK–Parkin Axis Negatively Regulates Necroptosis and Tumorigenesis by Inhibiting the Necrosome. Nat. Cell Biol..

[35] Otsubo K., Maeyashiki C., Nibe Y., Tamura A., Aonuma E., Matsuda H., Kobayashi M., Onizawa M., Nemoto Y., Nagaishi T. (2020). Receptor-Interacting Protein Kinase 3 (RIPK3) Inhibits Autophagic Flux during Necroptosis in Intestinal Epithelial Cells. FEBS Lett..

[36] Liu X., Liu L., Wang X., Jin Y., Wang S., Xie Q., Jin Y., Zhang M., Liu Y., Li J. (2023). Necroptosis Inhibits Autophagy by Regulating the Formation of RIP3/P62/Keap1 Complex in Shikonin-Induced ROS Dependent Cell Death of Human Bladder Cancer. Phytomedicine.

[37] Basgalupp S.P., Altmann V., e Vairo F.P., Schwartz I.V.D., Siebert M., Cravo R., Ribeiro E.M., dos Santos A.C., de Camargo Pinto L.L., Militão C.C. (2023). GBA1 Variants in Brazilian Gaucher Disease Patients. Mol. Genet. Metab. Rep..

[38] Kaech S., Banker G. (2006). Culturing Hippocampal Neurons. Nat. Protoc..

[39] Kholmukhamedov A., Schwartz J.M., Lemasters J.J. (2013). Mitotracker Probes and Mitochondrial Membrane Potential. Shock.

[40] Stirnemann J.Ô., Belmatoug N., Camou F., Serratrice C., Froissart R., Caillaud C., Levade T., Astudillo L., Serratrice J., Brassier A. (2017). A Review of Gaucher Disease Pathophysiology, Clinical Presentation and Treatments. Int. J. Mol. Sci..

[41] Pan B., Yang L., Wang J., Wang Y., Wang J., Zhou X., Yin X., Zhang Z., Zhao D. (2014). C-Abl Tyrosine Kinase Mediates Neurotoxic Prion Peptide-Induced Neuronal Apoptosis via Regulating Mitochondrial Homeostasis. Mol. Neurobiol..

[42] Baden P., Perez M.J., Raji H., Bertoli F., Kalb S., Illescas M., Spanos F., Giuliano C., Calogero A.M., Oldrati M. (2023). Glucocerebrosidase Is Imported into Mitochondria and Preserves Complex I Integrity and Energy Metabolism. Nat. Commun..

[43] Kim S., Wong Y.C., Gao F., Krainc D. (2021). Dysregulation of Mitochondria-Lysosome Contacts by GBA1 Dysfunction in Dopaminergic Neuronal Models of Parkinson’s Disease. Nat. Commun..

[44] Kiraly S., Stanley J., Eden E.R. (2025). Lysosome-Mitochondrial Crosstalk in Cellular Stress and Disease. Antioxidants.

[45] Adly A.A.M., Ismail E.A.R., Ibrahim F.A., Atef M., El Sayed K.A., Aly N.H. (2025). A 6-month Randomized Controlled Trial for Vitamin E Supplementation in Pediatric Patients with Gaucher Disease: Effect on Oxidative Stress, Disease Severity and Hepatic Complications. J. Inherit. Metab. Dis..

[46] Bendikov-Bar I., Maor G., Filocamo M., Horowitz M. (2013). Ambroxol as a Pharmacological Chaperone for Mutant Glucocerebrosidase. Blood Cells Mol. Dis..

[47] Higashi K., Sonoda Y., Kaku N., Fujii F., Yamashita F., Lee S., Tocan V., Ebihara G., Matsuoka W., Tetsuhara K. (2024). Rapid and Long-lasting Efficacy of High-dose Ambroxol Therapy for Neuronopathic Gaucher Disease: A Case Report and Literature Review. Mol. Genet. Genom. Med..

[48] Aries C., Köhn A., Täuber K., Rudolph C., Böttcher T., Bauer P., Fischer S., Muschol N. (2026). Exploring the Long-Term Use of Ambroxol in Gaucher Disease Type 2: Insights from Two Pediatric Cases. Front. Neurol..

[49] Dagda R.K., Zhu J., Kulich S.M., Chu C.T. (2008). Mitochondrially Localized ERK2 Regulates Mitophagy and Autophagic Cell Stress. Autophagy.

[50] Yamada T., Adachi Y., Fukaya M., Iijima M., Sesaki H. (2016). Dynamin-Related Protein 1 Deficiency Leads to Receptor-Interacting Protein Kinase 3–Mediated Necroptotic Neurodegeneration. Am. J. Pathol..

[51] Zhou H., Zhu P., Guo J., Hu N., Wang S., Li D., Hu S., Ren J., Cao F., Chen Y. (2017). Ripk3 Induces Mitochondrial Apoptosis via Inhibition of FUNDC1 Mitophagy in Cardiac IR Injury. Redox Biol..

[52] Arrázola M.S., Saquel C., Catalán R.J., Barrientos S.A., Hernandez D.E., Martínez N.W., Catenaccio A., Court F.A. (2019). Axonal Degeneration Is Mediated by Necroptosis Activation. J. Neurosci..

[53] Ren Y., Chen J., Wu X., Gui C., Mao K., Zou F., Li W. (2018). Role of C-Abl-GSK3β Signaling in MPP1-Induced Autophagy-Lysosomal Dysfunction. Toxicol. Sci..

[54] Marín T., Dulcey A.E., Campos F., de la Fuente C., Acuña M., Castro J., Pinto C., Yañez M.J., Cortez C., McGrath D.W. (2022). C-Abl Activation Linked to Autophagy-Lysosomal Dysfunction Contributes to Neurological Impairment in Niemann-Pick Type A Disease. Front. Cell Dev. Biol..

[55] Karim M.R., Liao E.E., Kim J., Meints J., Martinez H.M., Pletnikova O., Troncoso J.C., Lee M.K. (2020). α-Synucleinopathy Associated c-Abl Activation Causes P53-Dependent Autophagy Impairment. Mol. Neurodegener..

[56] Contreras P.S., Tapia P.J., González-Hódar L., Peluso I., Soldati C., Napolitano G., Matarese M., Heras M.L., Valls C., Martinez A. (2020). C-Abl Inhibition Activates TFEB and Promotes Cellular Clearance in a Lysosomal Disorder. iScience.

[57] Imam S.Z., Zhou Q., Yamamoto A., Valente A.J., Ali S.F., Bains M., Roberts J.L., Kahle P.J., Clark R.A., Li S. (2011). Novel Regulation of Parkin Function through C-Abl-Mediated Tyrosine Phosphorylation: Implications for Parkinson’s Disease. J. Neurosci..

[58] Ko H.S., Lee Y., Shin J.-H., Karuppagounder S.S., Gadad B.S., Koleske A.J., Pletnikova O., Troncoso J.C., Dawson V.L., Dawson T.M. (2010). Phosphorylation by the C-Abl Protein Tyrosine Kinase Inhibits Parkin’s Ubiquitination and Protective Function. Proc. Natl. Acad. Sci. USA.

[59] Harris K.G., Morosky S.A., Drummond C.G., Patel M., Kim C., Stolz D.B., Bergelson J.M., Cherry S., Coyne C.B. (2015). RIP3 Regulates Autophagy and Promotes Coxsackievirus B3 Infection of Intestinal Epithelial Cells. Cell Host Microbe.

[60] Song X., Li T. (2019). Ripk3 Mediates Cardiomyocyte Necrosis through Targeting Mitochondria and the JNK-Bnip3 Pathway under Hypoxia-Reoxygenation Injury. J. Recept. Signal Transduct..

[61] Rong Z., Zheng K., Chen J., Jin X. (2022). Function and Regulation of ULK1: From Physiology to Pathology. Gene.

[62] Turco E., Fracchiolla D., Martens S. (2020). Recruitment and Activation of the ULK1/Atg1 Kinase Complex in Selective Autophagy. J. Mol. Biol..

[63] Tian W., Li W., Chen Y., Yan Z., Huang X., Zhuang H., Zhong W., Chen Y., Wu W., Lin C. (2015). Phosphorylation of ULK1 by AMPK Regulates Translocation of ULK1 to Mitochondria and Mitophagy. FEBS Lett..

[64] Dorsey F.C., Rose K.L., Coenen S., Prater S.M., Cavett V., Cleveland J.L., Caldwell-Busby J. (2009). Mapping the Phosphorylation Sites of Ulk1. J. Proteome Res..

[65] Zachari M., Ganley I.G. (2017). The Mammalian ULK1 Complex and Autophagy Initiation. Essays Biochem..

[66] León R., Gutiérrez D.A., Pinto C., Morales C., de la Fuente C., Riquelme C., Cortés B.I., González-Martin A., Chamorro D., Espinosa N. (2023). C-Abl Tyrosine Kinase down-Regulation as Target for Memory Improvement in Alzheimer’s Disease. Front. Aging Neurosci..

[67] Chandía-Cristi A., Gutiérrez D.A., Dulcey A.E., Lara M., Vargas L., Lin Y.-H., Jimenez-Muñoz P., Larenas G., Xu X., Wang A. (2024). Prophylactic Treatment with the C-Abl Inhibitor, Neurotinib, Diminishes Neuronal Damage and the Convulsive State in Pilocarpine-Induced Mice. Cell Rep..

